# Neuronal biomolecular condensates and their implications in neurodegenerative diseases

**DOI:** 10.3389/fnagi.2023.1145420

**Published:** 2023-03-24

**Authors:** Jeongyeon Nam, Youngdae Gwon

**Affiliations:** Department of Molecular Cell Biology, Sungkyunkwan University School of Medicine, Suwon, Republic of Korea

**Keywords:** biomolecular condensates, liquid–liquid phase separation, phase transition, protein aggregation, neurodegenerative disease, synapse, RNA transport granule

## Abstract

Biomolecular condensates are subcellular organizations where functionally related proteins and nucleic acids are assembled through liquid–liquid phase separation, allowing them to develop on a larger scale without a membrane. However, biomolecular condensates are highly vulnerable to disruptions from genetic risks and various factors inside and outside the cell and are strongly implicated in the pathogenesis of many neurodegenerative diseases. In addition to the classical view of the nucleation-polymerization process that triggers the protein aggregation from the misfolded seed, the pathologic transition of biomolecular condensates can also promote the aggregation of proteins found in the deposits of neurodegenerative diseases. Furthermore, it has been suggested that several protein or protein-RNA complexes located in the synapse and along the neuronal process are neuron-specific condensates displaying liquid-like properties. As their compositional and functional modifications play a crucial role in the context of neurodegeneration, further research is needed to fully understand the role of neuronal biomolecular condensates. In this article, we will discuss recent findings that explore the pivotal role of biomolecular condensates in the development of neuronal defects and neurodegeneration.

## 1. Introduction

Compartmentalization is a critical feature that benefits eukaryotic cells, allowing them to concentrate functionally relevant biomolecules in specific subcellular organelles. Over time, eukaryotic cells have evolved to create distinct environments that promote the function of organelle-specific biomolecules, such as lysosomes or mitochondria. While membrane-bound organelles separated from the other cellular parts with lipid bilayers mainly composed of amphiphilic phospholipids have long been investigated, membrane-less assemblies of proteins and nucleic acids, defined as biomolecular condensates, have emerged as another paradigm to give rise to cellular organizations as a result of numerous studies over the last decade (Banani et al., 2017). De-mixed with the surrounding phase, biomolecular condensates embrace a subset of molecules of related functions, promoting many biological processes in themselves (Lyon et al., 2021). Now, the formation of biomolecular condensates is understood by the principle of liquid–liquid phase separation (LLPS).

Although biomolecular condensates are found in many eukaryotic cells, neurons, which differ structurally from other cells, exhibit a number of unique biomolecular condensates (Gopal et al., 2017; Milovanovic et al., 2018; Zeng et al., 2018; Wu et al., 2019; Mcdonald et al., 2020). Neurons are polarized cells with an axon that extends from the soma to transmit electrochemical information and multiple dendrites that receive inputs from adjacent neurons. In the interneuronal synapse, there are several microorganizations, including synaptic vesicle (SV) clusters, presynaptic active zones (AZ), and postsynaptic densities (PSD) (Feng et al., 2019; Emperador-Melero and Kaeser, 2020; Reshetniak and Rizzoli, 2021). Also, since axon and dendrites make up more than 90% of neuronal volumes, neurons face challenges in maintaining the structural and compositional integrity of synapses (Kasthuri et al., 2015). To overcome this, mRNAs encoding synaptic proteins are transported through neurite projections in a form of ribonucleoprotein (RNP) granule, or RNA transport granule, for localized translation (Das et al., 2021). Of note, LLPS plays a vital role in the assembly and functional regulation of these structures.

In this review, we will summarize recent updates about biomolecular condensates found exclusively in neurons. We will describe how multivalent interactions among components, facilitated by the principle of LLPS, generate biomolecular condensates. We will also discuss how the solidification of these condensates, from liquid to solid-like phase, is frequently found in the pathogenesis of neurodegenerative diseases. Additionally, we will describe the specialized roles of neuronal biomolecular condensates and their modification under disease-relevant contexts. This review will provide a comprehensive understanding of neuronal biomolecular condensates and their role in the mechanism of neurodegeneration.

## 2. Multivalency-driven LLPS As the molecular momentum to assemble biomolecular condensates

A de-mixing event work against the spontaneous increment in entropy to minimize the free energy in the system. Therefore, the contribution of enthalpy which offsets the loss of entropy is crucial for this de-mixing process as suggested by Flory-Huggins lattice models for polymer-solvent mixtures (Brangwynne et al., 2015; Nott et al., 2015; Workman and Pettitt, 2021). Enthalpic momentums are determined by the net change in intra-and intermolecular interactions during the de-mixing processes. Typically, for a given set of molecules to separate into a different phase, the sum of the magnitude of interactions among them exceeds the magnitude of interactions with other molecules. These molecules co-exist in two distinct phases following the de-mixing process of such solutions, one phase having a dense concentration and the other having a diluted concentration. LLPS is defined as a switch from 1-phase regime to 2-phase regime that results in a local enrichment of macromolecules above the saturation concentration in droplet-like structures (Hyman et al., 2014). In biological systems, these droplet-shaped foci composed mostly of proteins and nucleic acids that exhibit different phase properties from their surroundings are termed as biomolecular condensates (Banani et al., 2017; Lyon et al., 2021). The proteins in biomolecular condensates exhibit weak and transient interactions with multiple partners, which occur in both folded and intrinsically disordered regions (IDRs) (Banani et al., 2017; Shin and Brangwynne, 2017).

### 2.1. Folded domain

LLPS of sequentially positioned domains of one protein and short linear motifs (SLiM) of another protein is an example of how folded protein domains contribute to the valency-dependent assembly of biomolecular condensates (Hastings and Boeynaems, 2021). In the actin regulatory signaling system, SH3 domains of cytoplasmic protein NCK interact with proline-rich motifs, a prominent type of SLiM, of neuronal Wiskkott-Aldrich syndrome protein (N-WASP) (Li et al., 2012). Additionally, NCK binds to phosphorylated tyrosine residues of either Nephrin in kidney podocytes or SLP-76 in T cells, respectively through its SH2 domains (Li et al., 2012; Su et al., 2016). As the saturation threshold for phase separation in these signaling complexes, which are frequently represented as mesh networks, can be altered by varying the valency of connections, multivalency is crucial for encouraging protein condensation (Li et al., 2012; Banani et al., 2016; Su et al., 2016). Other examples of LLPS which require folded domain-mediated interactions include the LSm domain of Edc3 with leucine-rich motifs of Dcp2 and/or Pdc1 in p-bodies, and BTB and BACK domains of SPOP in nuclear speckles (Fromm et al., 2014; Bouchard et al., 2018).

### 2.2. Intrinsically disordered regions

Intrinsically disordered regions plays a crucial role for LLPS. IDRs do not have static tertiary structures and can be varied within a broad range of conformations whose energy states are similar to one another (Wright and Dyson, 2015). The conformational variance of IDR is often derived from the low complexity (LC) of its amino acid sequences (Uversky, 2002). Certain kinds of amino acids are enriched in IDRs depending on their chemical characteristics. Polar amino acids (e.g., serine and glutamine) and charged amino acids (e.g., glutamate, lysine, and arginine) are enriched in IDRs and contribute to LLPS driven by electrostatic interaction with other IDR-containing proteins or nucleic acids (Alberti et al., 2009; Pak et al., 2016). For example, the condensation of DDX4 in germ granules relies on the charged blocks in its IDR, and neutralizing the charges in these clusters inhibits the phase separation of DDX4 (Nott et al., 2015). Additionally, tyrosine and phenylalanine which have aromatic structures are frequently positioned in the IDR and enable IDR to have cation-π or π-π interactions required for LLPS, though tyrosine showed the better affinity with lysine or arginine compared to phenylalanine (Wang et al., 2018; Schuster et al., 2020). Otherwise, IDR could interact with SLiM as demonstrated by the interaction between the oligomerization domain of nucleophosmin (NPM1) and arginine-rich motifs of a group of nucleolar proteins in the granular component of nucleoli (Mitrea et al., 2016). Collectively, the sum of short-lived and weak interactions among biomolecules that happen simultaneously act as a driving momentum for LLPS.

## 3. Facilitation of protein fibrilization in the phase-deformed biomolecular condensates

Neurons become postmitotic during early development and typically remained differentiated for their entire lifespan, except in cases of cell cycle re-entry found in the brains of neurodegenerative diseases (Lee et al., 2009; Anda et al., 2016). Along with the closed nature of the brain accomplished by the limited molecular exchange across the blood–brain barrier, long-lived neurons often accumulate toxic protein fibrils and aggregates through the aging process. Therefore, it is important to study how soluble and functional proteins evolve into insoluble and dysfunctional aggregates to better understand the pathogenesis of neurodegenerative diseases (Soto and Pritzkow, 2018).

Nucleation-dependent fibrilization is a well-defined model to describe the formation of many biological polymers such as actin and tubulin cytoskeletons but also has long been thought to generate pathologic protein fibrils often found in the neurodegenerative diseases (Roychaudhuri et al., 2009). Misfolded proteins escaping from the proteostasis system often become the seed of this process (Hipp et al., 2019). Protein amyloids and filamentous protein aggregates with cross-beta structures are accumulated as insoluble deposits inside or outside of the cells. In particular, favorable environments for protein fibrilization are fostered in the nervous system with postmitotic neurons where seeds for amyloid formation can be enriched as aging proceeds (Hipp et al., 2019). Amyloid plaque and Lewy body found in the brains of Alzheimer’s disease (AD) and Parkinson’s diseases (PD), respectively, are well-known examples of protein amyloids.

Furthermore, recent studies have demonstrated that a liquid-to-solid phase transition is another route for building up the pathologic protein aggregates (Nedelsky and Taylor, 2019; Mathieu et al., 2020). Liquid-like biomolecular condensates become more dense and gel-like, and eventually transitioning into a solid-like phase during the pathogenic circumstances of human diseases (Alberti and Hyman, 2021). Especially, much evidence reflecting these pathologic transitions are readily identified in the process of neurodegenerative diseases including amyotrophic lateral sclerosis (ALS), frontotemporal dementia (FTD), huntington’s disease (HD), and tauopathy (Molliex et al., 2015; Patel et al., 2015; Lee et al., 2016; Ambadipudi et al., 2017; Jain and Vale, 2017; Peskett et al., 2018; Wegmann et al., 2018; Nedelsky and Taylor, 2019; Alberti and Hyman, 2021).

### 3.1. Amyotrophic lateral sclerosis–Frontotemporal dementia

**Figure 1:**
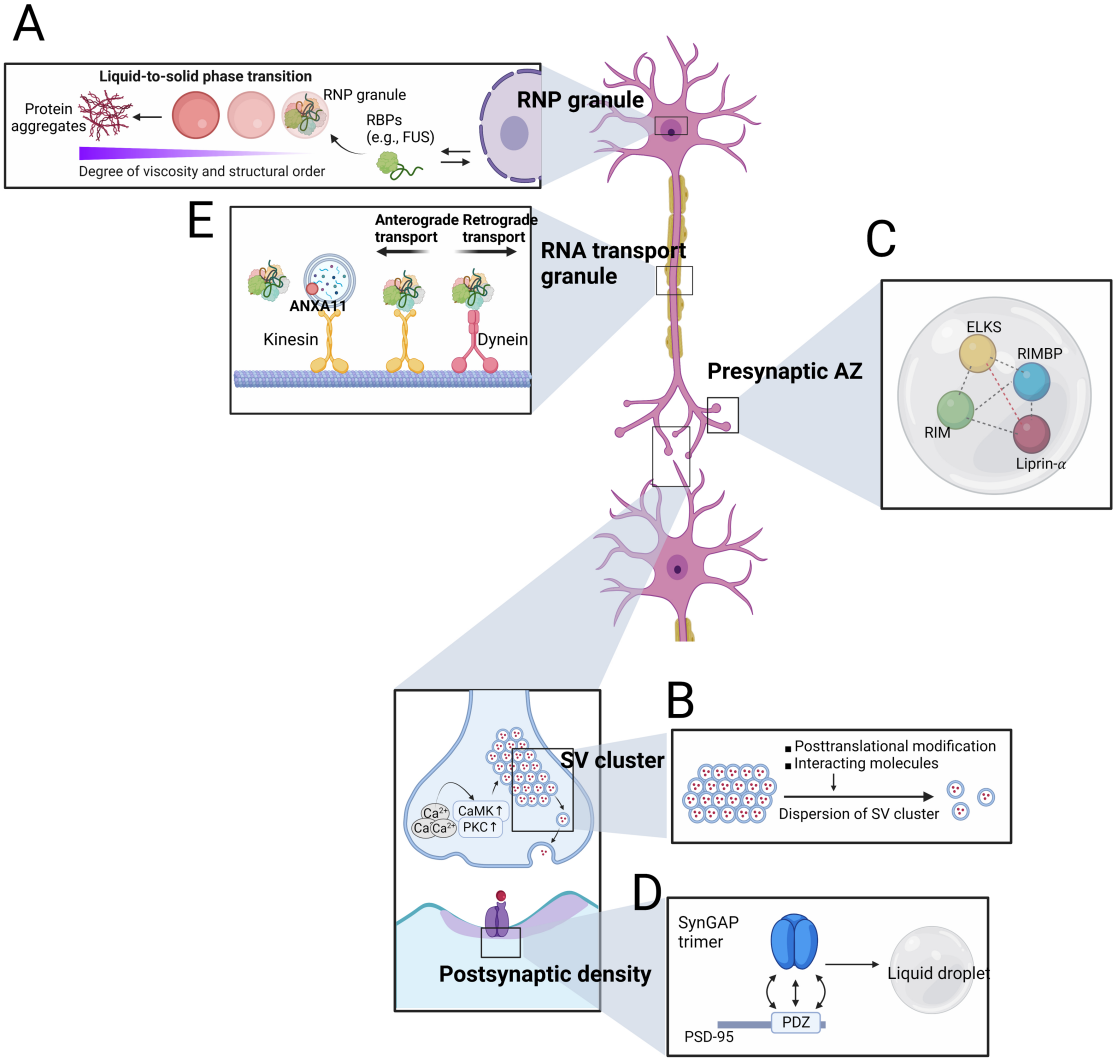
Neuronal condensates at the physiologic and pathologic contexts. **(A)** RNP granules, which are composed of mutant proteins encoded by amyotrophic lateral sclerosis (ALS)–frontotemporal dementia (FTD) causing genes, undergo a phase transition to solid states, resulting in the formation of protein aggregates. **(B)** Both posttranslational modification such as phosphorylation of synapsin by CaMKII dispersion and changes in interacting molecules (e.g., α-synuclein) lead to the dispersion of synaptic vesicle (SV) cluster. **(C)** Coexisting phase of scaffolding proteins serve as a hub to accommodate other presynaptic active zones (AZ) proteins. **(D)** The interaction between SynGAP trimer and PSD-95 plays a crucial role in assembling postsynaptic density. **(E)** RNA transport granules, either alone or with lysosomes, travel bi-directionally along the microtubule tract.

Amyotrophic lateral sclerosis–frontotemporal dementia, collectively now accounted as a spectrum disorder of ALS and FTD, is a representative research field where the phase transition from liquid to solid states of biomolecular condensates initiates the pathogenesis (Ling et al., 2013). Multiple genetic causes of ALS–FTD have been found in the genes encoding RNA binding proteins (RBPs) such as transactive response DNA-binding protein 43 kDa (TDP43), Fused in Sarcoma (FUS), HNRNPA1, and TIA1 which can undergo LLPS *in vitro* (Molliex et al., 2015; Patel et al., 2015; Mackenzie et al., 2017). Given that most RBP mutations causing ALS–FTD are located in IDR, it is not surprising that ALS–FTD-associated mutant FUS, HNRNPA1 and TIA1 exhibit a greater propensity to undergo LLPS at lower saturation concentration. Mutant proteins are also more likely to form denser, solid-like phases, and even insoluble fibrils (Figure 1A) (Molliex et al., 2015; Patel et al., 2015; Mackenzie et al., 2017).

Liquid-to-solid phase transition is also observed in the RNP granules, prominent types of biomolecular condensates. RNP granules are composed of RNA and RBPs and play a vital role in RNA regulation processes such as storage, splicing, decapping, and degradation. Wild-type TDP43, FUS, HNRNPA1, and TIA1 are present in RNP granules such as stress granules, p-bodies, and nucleoli and serve a general function. However, RNP granules harboring mutated RBPs had lower dynamicity, in accordance with *in vitro* experiments. Given that pathologic aggregates could be incubated in solidified RNP granules that offer an adequate niche for protein fibrilization, pathologic phase transition is necessary for the progression of ALS–FTD (Molliex et al., 2015; Patel et al., 2015; Zhang et al., 2019).

The expansion of GGGGCC hexanucleotide repeat in the first intron of *C9ORF72* is the most prevalent genetic cause of ALS–FTD (Dejesus-Hernandez et al., 2011; Renton et al., 2011). Though C9ORF72 protein levels are decreased in the patients with hexanucleotide expansion, the most striking feature is the accumulation of protein aggregates containing five dipeptide repeats (DPR), (GA)_n_, (GP)_n_, (GR)_n_, (PA)_n_, and (PR)_n_, which are synthesized by the repeat-associated non-AUG translation of GGGGCC repeat (Mori et al., 2013a,b; Saberi et al., 2018). Each DPR has a different level of toxicity. While (GP)_n_ and (PA)_n_ with uncharged and coiled structures are relatively non-toxic, (GR)_n_, and (PR)_n_ with the positively charged coiled structures are highly toxic with unique interactome (GA)_n_ is moderately toxic with compacted structure similar to the amyloid protein with cross-beta structure (Freibaum and Taylor, 2017). Arginine-containing (GR)_n_, and (PR)_n_ can separate with RBPs and RNA into droplets *in vitro*, and thus are distributed in RNP granules like nucleoli and stress granules (Kwon et al., 2014; Lee et al., 2016; Lin et al., 2016; Boeynaems et al., 2017; White et al., 2019). Furthermore, recruitment of (GR)_n_, and (PR)_n_ triggers the arrested dynamicity of RNP granules, leading to cellular toxicity (Lee et al., 2016; Boeynaems et al., 2017; White et al., 2019). Molecular findings related to the neuropathogenic role of arginine-containing DPRs have been studied in animal models, but more research is needed. (Choi et al., 2019; Cook et al., 2020).

Apart from the expedited protein fibrilization, phase transition in the disease-relevant context also affects the physiologic function of RNP granules. A lot of evidence supported that the function of biomolecular condensates is closely related to their material properties (Elbaum-Garfinkle et al., 2015; Zhu et al., 2019; Dorone et al., 2021; Lasker et al., 2022). ALS–FTD causing mutations also disrupts the function of RNP granules (Murakami et al., 2015). For example, ribosome biogenesis and concomitant translation rates are impaired by (GR)_n_ and (PR)_n_ DPR species that affect the integrating role of nucleophosmin in the LLPS of a nucleolar network (White et al., 2019).

Cells have regulatory mechanisms to eliminate aberrant RNP granules with altered material properties because they are associated with cellular dysfunction and toxicity. Protein quality control (PQC) systems such as chaperone, unfoldase, proteasome, and autophagy systems are involved in disassembling or clearing aberrant RNP granules (Buchan et al., 2013; Ganassi et al., 2016; Turakhiya et al., 2018; Wang et al., 2019; Gwon et al., 2021). It is worth noting that another functional category of ALS–FTD-causing genes, other than RBPs, is PQC, including *VCP*, *SQSTM1*, *OPTN*, and *UBQLN2*. Among them, de-mixing of SQSTM1 and UBQLN2 in both cell-free and cellular systems were identified and mutations linked to ALS–FTD lead to reduced fluidity (SQSTOM1) and even liquid-to-solid phase transition (UBQLN2) (Dao et al., 2019; Faruk et al., 2021). Mutated VCP gene is associated with persistent stress granules lacking reversibility and is implicated in the disease-relevant phenotypes (Buchan et al., 2013).

### 3.2. Huntington’s disease

Huntington’s disease is caused by the accumulation of mutant huntingtin proteins with a polyglutamine (poly Q) tract. The expansion of the CAG trinucleotide in the first exon of *HTT* gene is responsible for this abnormal protein expression. Protein products of the first exon of *HTT* separate into liquid droplets when the number of glutamine repeats is less than those in HD patients (Peskett et al., 2018). As the poly Q length increases, HTT exon 1 protein assemblies are converted to solid-like structures, indicating that phase transition is the underlying process of how the pathogenic HTT protein species exert cellular toxicity (Peskett et al., 2018). Molecular factors such as protein methylation and chaperone function affect the phase behavior of HTT protein (Aktar et al., 2019; Ratovitski et al., 2022). It will also be intriguing to investigate the crosstalk between HTT condensates and other biomolecular condensates in the development of HD (Sønmez et al., 2021).

### 3.3. Tauopathy

Neurofibrillary tangles, or intra-neuronal tau deposits, are present in various neurodegenerative diseases such as AD, progressive supranuclear palsy, FTD, and parkinsonism linked to chromosome 17 (FTDP-17) (Gøtz et al., 2019). The progression of tauopathies is associated with the instability of microtubules, which occurs in tandem with the toxicity of pathologic conformations of tau, from oligomers to fibrils (Zhang et al., 2022). Recent studies have illustrated several structural and pathogenic factors governing tau LLPS (Boyko and Surewicz, 2022). First, an amphoteric characteristic of tau possessing a negatively charged N-terminal part and positively charged middle and C-terminal part allows for electrostatic interactions with other tau molecules (homotypic interaction), other proteins, and RNA (Zhang et al., 2017; Boyko et al., 2019; Ukmar-Godec et al., 2019; Siegert et al., 2021). Acetylation on lysine residues neutralizes the charge of tau, perturbing tau-RNA interactions and reducing LLPS propensity (Ukmar-Godec et al., 2019). Second, modification associated with tauopathies affects the LLPS behavior of tau. Both P310L mutation found in FTDP-17 and pathogenic phosphorylation promote phase separation and generate tau droplets with less dynamicity (Ambadipudi et al., 2017; Wegmann et al., 2018; Kanaan et al., 2020). Third, alternative splicing on exon 10 of tau producing fewer microtubule-binding repeats inhibits tau LLPS (Ambadipudi et al., 2017). Fourth, tau and microtubule/microtubule-associated proteins reciprocally regulate in the separated phase, linking to functional aspects of tau (Hernãndez-Vega et al., 2017; Siahaan et al., 2019; Tan et al., 2019; Zhang et al., 2020; Savastano et al., 2021). Overall, the pathologic phenotypes of tauopathies are influenced by differential tau LLPS depending on normal or disease-relevant context.

Apart from the homogenous tau condensates, the interaction of tau with RBPs from the proteomic analysis and recruitment of tau to stress granules were revealed (Vanderweyde et al., 2016). Among these RBPs, TIA1 potentiates tau LLPS, and loss of TIA1 ameliorates the neurodegeneration in P301S mutant tau-expressing mice (Apicco et al., 2018; Ash et al., 2021). Tau condensation through TIA1 is understood as the molecular basis for facilitating tau oligomerization and causing neurodegeneration (Jiang et al., 2019; Ash et al., 2021). Additionally, tau aggregates ensemble with small nuclear RNAs and small nucleolar RNAs are found in serine arginine repetitive matrix protein 2 (SRRM2)-positive nuclear splicing speckle and required for alterations in splicing process (Lester et al., 2021). Therefore, reciprocal regulation between tau and biomolecular condensates underlies much of tauopathies.

### 3.4. Synucleinopathy

Aberrant accumulation of α-synuclein is a hallmark of several neurodegenerative diseases including Parkinson’s disease, dementia with Lewy bodies, and multiple system atrophy, collectively characterized by motor symptoms including tremor, rigidity, bradykinesia, and posture instability and cognitive impairments (Magalhåes and Lashuel, 2022). Recent research has shown that α-synuclein undergoes phase separation, which is connected to its aggregation (Ray et al., 2020). α-synuclein liquid droplets lose their dynamicity after prolonged incubation or when familial mutations of PD are introduced. Also, cellular α-synuclein condensates are converted to a solid-like phase and further evolve into the aggresome upon the addition of metal ion, which promote the aggregation of α-synuclein. α-synuclein LLPS occurs through homotypic interactions among non-amyloid-β component (NAC) domains, but intramolecular interactions between the N-terminal region and C-terminal tails masks the NAC domain under physiologic conditions, making the protein soluble (Sawner et al., 2021). Certain changes in the milieu, including pH and ionic composition, can convert the conformational state of α-synuclein and trigger phase separation, further leading to aggregation.

## 4. Synaptic vesicle cluster, presynaptic active zone, and postsynaptic density: Biomolecular condensates in synaptic connection

Synapses are the connection between presynaptic and postsynaptic neurons, and are composed of a presynaptic terminus, synaptic cleft, and postsynaptic dendritic spine, each enriched with distinct profiles of proteins according to their functions. The presynaptic axon terminus has SV clusters as an inventory of SV and triggers the release of neurotransmitters at the presynaptic active zone (AZ). Proper assembly of SV cluster and presynaptic AZ is governed by LLPS, which implies that they are biomolecular condensates (Milovanovic et al., 2018; Wu et al., 2019; Mcdonald et al., 2020). Understanding synaptic LLPS would give a unique viewpoint on neuropathies because cognitive dysfunctions are easily identified in the brains of neurodegenerative disease patients at the synapse level.

### 4.1. SV cluster

The SV cluster is a subsynaptic structure where dozens to thousands of SVs are closely placed near the presynaptic AZ without a boundary (Reshetniak and Rizzoli, 2021). In response to releasing stimuli, such as Ca^2+^ influx that can be translated into the activation of calcium/calmodulin-dependent protein kinase family or protein kinase C (PKC), SV exocytosis results in the release of neurotransmitters to the synaptic cleft (Cesca et al., 2010). The spatial confinement of SVs within SV cluster remained elusive until LLPS of synapsin, the major constituent of SV, was discovered (Milovanovic et al., 2018). The IDR of synapsin is essential for this process by recruiting SH3 domain-containing proteins to synapsin condensates. Furthermore, phosphorylation of synapsin by CaMKII disperses the synapsin condensates, recapitulating the dissolution event of SVs from the cluster (Figure 1B). Abrogation of synapsin LLPS by adding an IDR-targeting antibody disrupts SV clusters in the lamprey, supporting the crucial role of synapsin LLPS in organizing SV clusters (Pechstein et al., 2020). While synapsin condensates coexist with liposome *in vitro*, cation-π interaction with SV membrane protein synaptophysin potentiates the SV organizing ability based on the observation of SV-like clusters even in the fibroblast cells (Kim et al., 2021; Park et al., 2021). Another partner of synapsin condensates is α-synuclein, and excessive α-synuclein to synapsin reduces the synapsin condensate formation (Hoffmann et al., 2021). Because α-synuclein and synapsin are functionally linked in terms of SV regulation, further investigation of their relationship in PD contexts, in which α-synuclein is accumulated, will provide a novel insight of PD pathogenesis (Kramer and Schulz-Schaeffer, 2007; Atias et al., 2019; Bridi et al., 2021).

### 4.2. Presynaptic AZ

The Presynaptic AZ is located beneath the presynaptic membrane and is where SV exocytosis occurs (Sþdhof, 2012). Presynaptic AZ is seen as dense marks under electron microscopy and has a concentrated proteome, making them distinct from the surroundings. Scaffold proteins such as Glutamine/leucine/lysine/serine-rich protein (ELKS), Liprin-α, Rab3-interacting molecule (RIM), and RIM-binding protein (RIMBP) interact with each other during SV fusion events to recruit other AZ proteins including voltage-gated Ca^2+^ channel (VGCC) and cell adhesion molecules (Sþdhof, 2012). Recent reports have demonstrated that these scaffold molecules undergo LLPS dominated by both structured domain-and IDR-affiliated multivalent interactions (Wu et al., 2019; Mcdonald et al., 2020; Liang et al., 2021). *In vitro* reconstitution systems have revealed co-phase separation of RIM and RIMBP, driven by the multivalent interactions between the proline-rich motif of RIM and SH3 domain of RIMBP. This event is further coupled to the incorporation of VGCCs into their separated phase (Figure 1C).

Liprin-α plays a pivotal role in regulating AZ condensates profiles. Oligomeric Liprin-α assembled upon a coiled-coiled region can be de-mixed with ELKS (Liang et al., 2021). ELKS and Liprin-α condensates were identified in the developing synapses of *Caenorhabditis elegans* (Mcdonald et al., 2020). Interestingly, Liprin-α determines whether RIM, RIMBP, and VGCC are distributed with ELKS condensates (Liang et al., 2021). ELKS-Liprin-α coexisting droplets accommodate RIM and RIMBP, while liquid droplets composed of ELKS alone cannot recruit both. However, RIM and RIMBP in the ELKS-resident droplets failed to incorporate VGCC. The heterogeneity between the condensates provides the molecular basis for distinct protein–protein interaction networks found in the synapse (Lautenschlæger, 2022).

### 4.3. Postsynaptic densities

Similar to the presynaptic AZ, PSD is an electron-dense area at the postsynaptic membrane proximal to AZ (Sþdhof, 2012). PSD contains a significant number of proteins that respond to the released neurotransmitters, including glutamate receptors, downstream signaling molecules, and scaffold proteins (Feng and Zhang, 2009). LLPS also plays an essential role in the assembly of PSD. The multivalent interaction between the trimeric complex of Ras/Rap GTPase-activating protein SynGAP and PDZ domain of postsynaptic density protein 95 (PSD-95) forms liquid-like droplets (Figure 1D). These two highly abundant proteins act on the normal structure and function of the postsynaptic neuron (Vazquez et al., 2004; Chen et al., 2015; Zeng et al., 2016). Replacing endogenous SynGAP with a trimerization-defective mutant SynGAP or endogenous PSD-95 with mutant PSD-95 that fails to interact with SynGAP reduces spine volume and postsynaptic condensates, underscoring the functional role of SynGAP/PSD-95 condensates in regulating postsynaptic activity.

Phase separation also contributes to the differential assembly of excitatory PSD (ePSD) and inhibitory PSD (iPSD) (Zeng et al., 2018). In the supported membrane bilayer system, co-phase separation of four enriched scaffold proteins of ePSD, PSD-95, guanylate kinase-associated protein (GKAP), SH3 and multiple ankyrin repeat domains protein (Shank), and Homer scaffold protein (Homer), was achieved. These condensates also recruit SynGAP and N-methyl D-aspartate receptor subtype 2B (NR2B) but repel gephyrin, a key scaffold of iPSD. Therefore, ePSD formation by LLPS enables an augmented glutamate receptor response in a condensed area. On the contrary, iPSD is formed by the LLPS of gephyrin and glycine or GABA receptors (Bai et al., 2021).

## 5. RNA transport granule relays RNA and RBP to neurites for localized translation

Ribonucleic acid (RNA) transport granules are specialized ribonucleoprotein granules found in neurons that transport mRNA to axons and dendrites for localized translation (Fernandopulle et al., 2021). In order to travel to the proximal parts of neuron, they rely on microtubule-dependent transport machinery which bi-directionally dispatch biomolecules along with the track assembled with microtubules by aid of motor proteins such as dynein and kinesin (Abouward and Schiavo, 2021). Recent findings has suggested that functional failures in RNA transport granules are caused by mutations in RBPs that are genetically associated with neurodegenerative diseases (Figure 1E) (Liao et al., 2019; Fernandopulle et al., 2021).

One such RBP is Annexin A11 (*ANXA11*), which is a genetic risk for both familial and sporadic cases of ALS–FTD (Smith et al., 2017). Mutations in ANXA11 result in impaired Ca2+ homeostasis and protein translation, as well as hinder proper elimination of stress granules (Nahm et al., 2020). In the neuronal context, ANXA11 is found in the proteomes labeled in the vicinity of both lysosome and RNA transport granules and links them during the cargo transport (Liao et al., 2019). Since ANXA11 proteins mutated in annexin repeats region less interacts with the lysosome, the travel distances of RNA cargo in *ANXA11* mutation harboring neurons are reduced as well.

Another cause of the functional impairment of RNA transport granules is mutations in TAR DNA-binding domain (*TARDBP*) that result in ALS–FTD and code for TDP-43. In addition to the pathogenic aggregation of TDP-43, its physiologic functions that regulate gene expression in both transcriptional and translational levels are also affected. Cellular dysfunctions such aberrant splicing and abnormal RNP granules are brought on by the loss of TDP-43 functions and increased cytoplasmic localizations that are related to changes in the interplay between the proteome and transcriptome (Tollervey et al., 2011; Ling et al., 2015). In addition, TDP-43 directs RNA trafficking to synaptic processes in a microtubule-dependent manner (Alami et al., 2014). RNA transport granules with TDP-43 demonstrate anterograde transport, whereas those without TDP-43 undergo retrograde transport. Moreover, a kymograph from fruit fly motor neurons expressing TDP-43 A315T or M337V mutants showed RNA transport granules travel retrogradely, causing the cytoplasmic accumulation of TDP-43 which are reminiscent of ALS histology (Neumann et al., 2006; Alami et al., 2014). Moreover, the loss of TDP-43 results in poor local translation, which compromises synaptic transmission. (Diaper et al., 2013; Wong et al., 2021).

## 6. Conclusion and future perspectives

Upon the dramatic shift in the distribution of biomolecules between mixed and de-mixed states accomplished by weak and multivalent interactions, biomolecular condensates are understood as functional units of biological phenomena. Multiple pathogenic factors including genetic risks of neurodegenerative disease or cellular stresses alter the material properties and dynamicity of biomolecular condensates. In the neuronal context, fibrilization of pathogenic proteins are promoted at aberrant biomolecular condensates where phase transition occurs. In addition, the phase transition of biomolecular condensates is often involved in their loss of function. There is still much to be understood because the research on neuronal condensates is still in its early stages. This work will unveil how abnormal alteration of neuronal condensates triggers neuronal dysfunction such as impaired axonal transport, loss of synaptic plasticity, and excitotoxicity. Many genetic loci for synaptic proteins that are identified as neurodegenerative disease-causing variants can be attractive topics in that a considerable portion of disease-associated genes encode condensate-forming proteins (Stein et al., 2010; Liu et al., 2021; Prokopenko et al., 2021; Banani et al., 2022). Furthermore, it will be crucial to reflect the actual pathophysiology of neurodegenerative disease by establishing a higher order system than *in vitro* and cellular levels using multicellular or animal models to analyze the material properties and dynamics of biomolecular condensates. Those approaches will broaden our knowledge and be utilized as a bridge to develop therapeutic interventions from molecular findings.

## Author contributions

JN and YG wrote the manuscript. YG conceived and revised the manuscript. All authors contributed to the article and approved the submitted version.

## Funding

This work was supported by the National Research Foundation of Korea (NRF) grant funded by the Korea government (MSIT) (No. 2022R1C1C1012534).

## Conflict of interest

The authors declare that the research was conducted in the absence of any commercial or financial relationships that could be construed as a potential conflict of interest.

## Publisher’s note

All claims expressed in this article are solely those of the authors and do not necessarily represent those of their affiliated organizations, or those of the publisher, the editors and the reviewers. Any product that may be evaluated in this article, or claim that may be made by its manufacturer, is not guaranteed or endorsed by the publisher.
